# Supplementary material for: The prevalence and risk factors associated with Iron, vitamin B12 and folate deficiencies in pregnant women: A cross-sectional study in Mbeya, Tanzania
Source: PLOS Glob Public Health. 2023 Apr 21;3(4):e0001828. doi: 10.1371/journal.pgph.0001828 (PMC10121015; doi:10.1371/journal.pgph.0001828)
Supplement: Attachment — Submitted filename: A rebuttal letter_Sauli_FINALE2023.docx [file pgph.0001828.s001.docx]

Dear Dickson Abanimi Amugsi, PhD

Thank you very much for giving us the opportunity to submit a revised draft of our manuscript titled “The prevalence and risk factors associated with the deficiencies of Iron, Vitamin B12 and Folate among pregnant women attending antenatal clinics in Mbeya Tanzania”
We appreciate the time and effort that you and reviewers have dedicated to providing valuable feedback on our manuscript.

Here is a point-by-point response to the journal requirements, and reviewers’ comments and concerns.

**Journal Requirements**

**Comment 1:** *In the ethics statement in the Methods, you have specified that verbal consent was obtained. Please provide additional details regarding how this consent was documented and witnessed, and state whether this was approved by the IRB.*

**Response**: Thank you very much for this comment. Only written consent was thought, and we have made corrections (line number 262-268).

**Comment 2:**

*Please amend your detailed Financial Disclosure statement. This is published with the article. It must therefore be completed in full sentences and contain the exact wording you wish to be published.*

*a. Please clarify all sources of funding (financial or material support) for your study. List the grants (with grant number) or organizations (with url) that supported your study, including funding received from your institution. 
b. State the initials, alongside each funding source, of each author to receive each grant.
c. State what role the funders took in the study. If the funders had no role in your study, please state: “The funders had no role in study design, data collection and analysis, decision to publish, or preparation of the manuscript.”
d. If any authors received a salary from any of your funders, please state which authors and which funders.*

*If you did not receive any funding for this study, please simply state: “The authors received no specific funding for this work.”*

**Response:** We highly appreciate comment and, we have rephrased the financial disclosure statement (line number 450).

**Comment 3:** *In the Funding Information you indicated that no funding was received. Please revise the Funding Information field to reflect funding received.*

**Response:** Thank you very much for this comment. No specific funding was received for this study (line number 450).

**Comment 4:** *Your manuscript is missing the following sections: Introduction. Please ensure these are present, and in the correct order, and that any references to subheadings in your main text are correct. An outline of the required sections can be consulted in our submission guidelines here:* *https://journals.plos.org/globalpublichealth/s/submission-guidelines#loc-parts-of-a-submission*

**Response:** Thank you very much and, we highly appreciate the insightful and helpful comments. We have made correction and the introduction section is now clearly indicated (line number 72).

**Additional Editor Comments**

**Comment**: *Thank you for submitting your work to PGPH for publication. Two independent reviewers have reviewed your manuscript and recommended minor revision. However, I have noticed that the issues they raised are quite substantial, and will need some amount of effort to address. I suggest you diligently address their comments and resubmit your manuscript for consideration. Also, carefully proofread the entire manuscript to address possible grammar and diction issues.*
**Response:** We appreciate the time and effort that you and reviewer have dedicated to providing valuable feedback on our manuscript. Thank you very much.

**Review Comments to the Author**

**Reviewer #1**

**Comment 1**: *TITLE
1. Clarify if the study focuses on one or all three of the deficiencies, Iron, Vitamin B12 and Folate.
2. Consider including a statement in the title about the geographical focus of the study (e.g. Mbeya, Tanzania).
3. Consider adding a phrase such as "a cross-sectional study" to provide additional information about the study design.*
 **Response:** We agreed with this comments and rephrased the title as suggested (line number 1-2).

**Comment 2:** *ABSTRACT
1. I recommend that the authors provide more detail regarding the methodology used in the study, such as the sampling technique and the specific dietary patterns that were assessed.
2. The authors should provide more clarity on the interpretation of the results. For example, it would be helpful to provide more information on how the different factors were associated with the deficiencies, and what implications these findings have for pregnant women in Tanzania.
3. The authors should consider extending their discussion to include implications for policy and public health interventions.*

**Response:**

1. Thank you very much and we have re-write the paragraph as suggested (line number 222-239).
2. We highly appreciate the insightful and helpful comments. We have rephrased results section as suggested (line number 277-328).
3. We have rephrased the discussion section as suggested (line number 407-411)

**Comment 3**: *INTRODUCTION
I recommend that the authors should provide
1. More information about the prevalence of iron, vitamin B12, and folate deficiencies in other countries and regions, as well as a comparison to the prevalence in Tanzania.
2. A more detailed description of the potential health risks associated with deficiencies in iron, vitamin B12, and folate, including both maternal and neonatal risks.
3. More information about the current recommendations for vitamin B12 supplementation during pregnancy in Tanzania and the potential benefits of supplementing with vitamin B12.*

**Response:**

1. Thank you very much and we agreed with comment. We have added more information on the prevalence of iron, vitamin B12, and folate deficiencies as suggested (line number 105-125)
2. We agreed with this comments and rewrite the introduction section to include description of the potential health risks associated with deficiencies in iron, vitamin B12, and folate, including both maternal and neonatal risks (line number 72-126).
3. We agreed with this comments and added information on lack of recommendations for vitamin B12 supplementation and the potential benefits of supplementing with vitamin B12 (line number 112-125).

**Comment 4**: *METHODS
Sampling and data collection procedure
The authors should
1. Provide more information on the Lwanga and Lemeshow formula and its relevance to the study.
2. Provide further explanation on the two-stage cluster sampling used in the study.
3. Discuss the implications of the reduced sample size on the results of the study.
4. Ensure that the operations manual for blood collection, handling, and transportation of blood and urine samples is detailed and comprehensive.
5. Also provide more information on the exact procedures used for cell lysate sample preparation and storage.
6. Provide further information on the temperature and duration of the transport of the samples to the Tanzania Food and Nutrition Centre (TFNC) laboratory.
7. Should include more information on how the samples were handled and processed at the TFNC laboratory.
Study tool
1. It would be beneficial for the authors to provide more information on the translation process. This could include information on the qualifications of the translators and the methods used to check the accuracy of the translation.
2. The authors should consider making the questionnaire available in the paper for readers to review.
3. The authors should state the response categories for each question more clearly.
4. The authors should consider adding information on how they ensured the reliability and validity of the questionnaire within the context of their study and environment.*

**Response**:

*Sampling and data collection procedure*

1. More information on the Lwanga and Lemeshow formula and its relevance to the study has been added (line number 147-149).
2. We agreed with this comments and more explanation on the two-stage cluster sampling has been added (line number 151-165).
3. We have added the information regard the implications of the reduced sample size on the results of the study (line number 149-151).
4. A detailed and comprehensive operation manual for blood collection, handling, and transportation of blood and urine samples (line number 166-198).
5. We agreed with this comment and more information has been added as suggested (line number 184-192).
6. We agreed with this comment and more information has been added as suggested (line number 194-196).
7. We agreed with this comment and more information has been added as suggested (line number 196-198).

*Study tool*

1. We agreed with this comment and more information has been added as suggested (line number 218-226 & 264-271).
2. The questionnaires and data set are available in the public repository: <https://osf.io/7ysb9./>
3. We agreed with this comment and more information has been added as suggested (line number 229-234).
4. Thank you very much for pointing this out and we have added information on reliability as suggested (line number 264-271).

**Comment 5:** *RESULTS
The authors have done a thorough job of presenting the data collected from their study. However,
1. It would be beneficial to provide more detailed descriptions of the socio-economic, demographic, and dietary characteristics of the study participants.
2. Provide information on the potential confounding factors that may have influenced their findings.*

**Response:**

1. More information on the descriptions of the socio-economic, demographic, and dietary characteristics of the study participants (line number 273 -298).
2. We agreed with this comment and more information has been added (line number 252-256 & 299-301).

**Comment 6**: *DISCUSSION
The authors should consider
1. Expanding on the implications of their findings and discussing potential strategies to address the identified deficiencies among pregnant women.
2. Exploring the potential benefits of multiple micronutrients supplementation, as well as the potential long-term implications of deficiencies during pregnancy.
3. Contextualizing their findings and exploring the potential differences in prevalence and associated risk factors across different regions of the country.*

**Response:**

1. Thank you very much and we have rewritten the discussion section as suggested (line number 325 -410).
2. The potential benefits of multiple micronutrients supplementation (line number 406 410)
3. Thank you very much for pointing this out. We agreed the comment and added more information as suggested (line number 352-355 & 371 -376).

**Reviewer #2
Comment 1:** *According to the guidelines of PLOS, the author is supposed to provide numbering for each line sentence of the manuscript to easy referencing by the reviewer. This was not provided. Consider incorporating the line numbering.*

**Response:** Thank you very much and we have inserted the line numbering throughout the manuscript.

**Comment 2**: *Rephrase the sentence for clarity in the methodology: …Mbeya is located in the southern highlands of Tanzania and lies at an altitude of 475metres above sea level with high peaks of 2981metres above sea level at Rungwe district higher attitudes*

**Response**: Thank you very much and, we have rephrased the sentence as suggested (line number 133).

**Comment 3**: *Refer to the sentence on the methodology “Precisely, 100ml of whole blood in each EDTA tube was collected and mixed with ascorbic acid to make cell lysate samples for RBC folate determination”*

**Response**: We agreed with this suggestion and rephrased the sentence as suggested (line number 183-188).

**Comment 4**: *Address the references sequencing. See reference [12] then [14], then [13], [43] then [15]*.

**Response:** Thank you very much and we agreed with this comment and address the references sequencing.

**Comment 5**: *Insert reference: Ethical clearance para: All procedures followed were per the ethical standards of the Helsinki Declaration of 1975*

**Response**: We have inserted the reference as suggested (line number 263).

**Comment 6**: *In the results part, rephrase the sentence: As presented in table 2, about one-quarter (24.7%) of the participants consumed two times dark leafy green vegetables daily among whom 16.8%, 34.0% and 24.0% had serum vitamin B12, ferritin and, folate deficiencies respectively.*

**Response:** We have rephrased the sentence as suggested (line number 293-295).

**Comment 7**: *A systemic review of vitamin B12 deficiency among pregnant women in all trimesters documented prevalence in a range of 20% - 30% [39]. In contrary, our study found a low prevalence of vitamin B12 deficiency among pregnant women of gestation period less than 28 weeks*

**Response:** We agreed with comments and rewrite the paragraph (line number 352-355).

**Comment 8**: *Studies have showed that the optimal folate required is essential during pregnancy to encounter the growing demands of both the mother and the fetus [26, 32].*

**Response**: We agreed with this suggestion and rephrased the paragraph as suggested (line number 371-373).

**Comment 9**: *In conclusion para it is not clear rephrase it… We believe that our results represent the profile of pregnant women with gestation period of below 28 weeks and, as the measurements were performed in 420 subjects using laboratory methods considered suitable for analysis; …(it clearly brings out what new findings…, that affirms what known or unknown massage to the reader?). Furthermore, our results enable the identification of key risk factors of developing folate, iron and vitamin B12 deficiencies.*

**Response:** We highly appreciate the insightful comments and rephrased the sentence as suggested (line number 412-415).

**Comment 10:** *In Tanzania and many developing counties, much effort has been given to iron and folic acid supplementation during pregnancy, but less emphasis has been given to vitamin B12 supplements*

**Response:** We agreed with comment and rephrased the sentence (line number 412-415).

**Comment 11**: *Rephrase this sentence in the conclusion: It is recommended that future studies longitudinal should explore the risk factors of serum folate, ferritin and vitamin B12 deficiencies among pregnant women*

**Response:** Thank you very much and we have rephrased the sentence (line number 421- 423).

Sincerely,

Ray Masumo, PhD
